# ChemLint: Conversational Cheminformatics with Large Language Models

**DOI:** 10.1021/acs.jcim.6c00638

**Published:** 2026-08-28

**Authors:** Derek van Tilborg, Francesca Grisoni

**Affiliations:** Institute for Complex Molecular Systems (ICMS), Department of Biomedical Engineering, 425625 Eindhoven University of Technology, P.O. Box 513, 5600 MB Eindhoven, The Netherlands

## Abstract

We introduce ChemLint, an open-source Model Context Protocol (MCP) server that connects any MCP-compatible large language model to a curated suite of local cheminformatics and machine learning tools, enabling rigorous molecular data handling through a conversational interface. Molecular machine learning studies are frequently undermined by inconsistent data preprocessing, including invalid SMILES, unresolved duplicates, and train-test leakage, yet existing large language model (LLM)-based chemistry tools do not address these data-centric challenges. ChemLint provides tools for data exploration and diagnostics, molecule standardization, and machine learning modeling. All operations are executed deterministically by established libraries and recorded in a project manifest that traces every action, supporting reproducibility and making curation choices explicit. We demonstrate through several examples how ChemLint can be used to identify common data quality issues, evaluate splitting strategies, and execute complete modeling pipelines from raw data to evaluation. ChemLint is freely available at https://github.com/molML/ChemLint.

## Graphical abstract

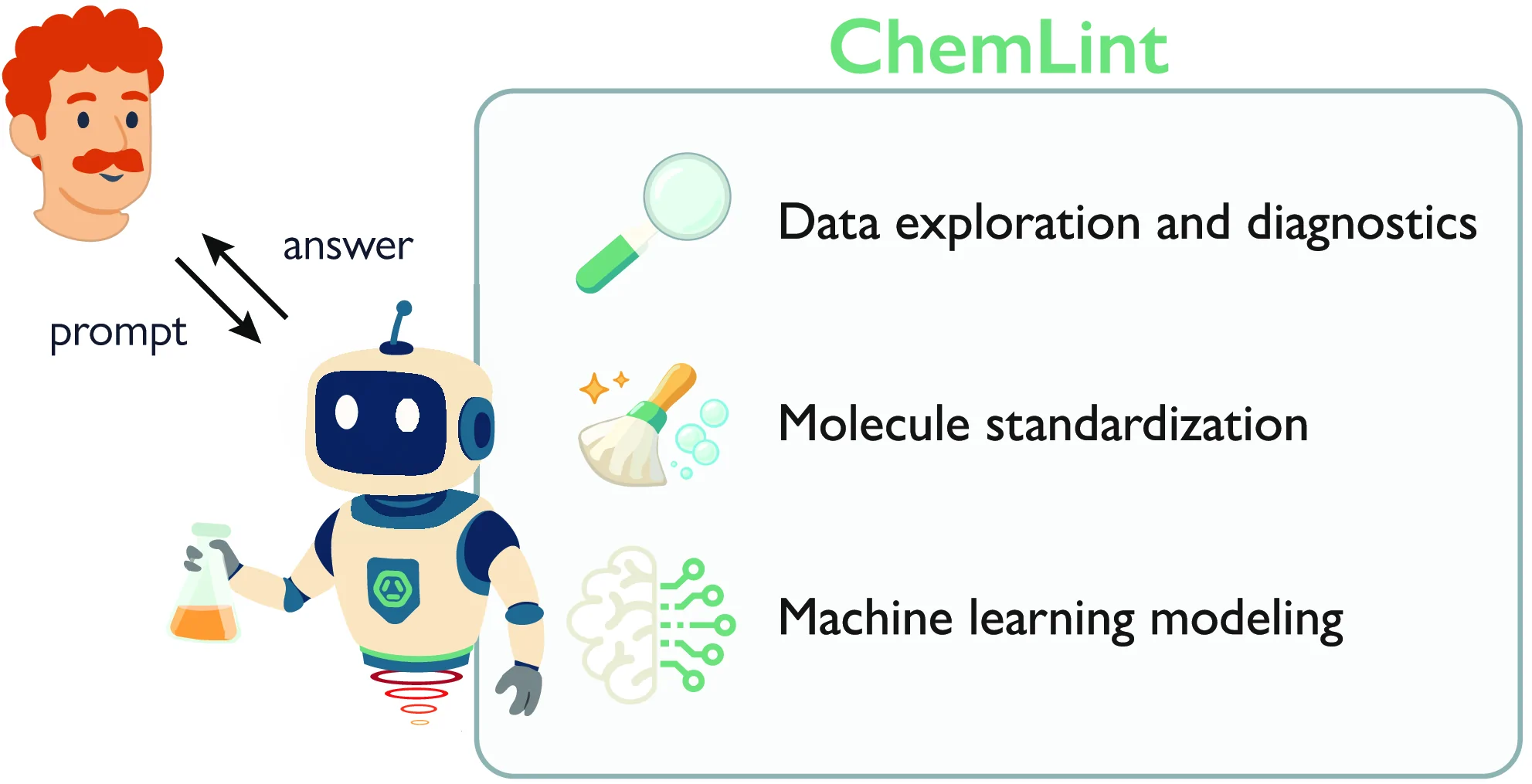

## Introduction

Machine learning is increasingly used to accelerate drug discovery. However, molecular machine learning suffers from a pervasive data quality problem that compromises model evaluation and reproducibility.^1–3^ In practice, differences in preprocessing can change model performance estimates and lead to conclusions that do not transfer beyond a specific curation pipeline.^4,5^ This might undermine the confidence in published molecular machine learning research and hold back progress toward useful models for drug discovery. Even widely cited benchmark data sets^6,7^ contain fundamental errors such as chemically invalid molecules or artifacts such as the presence of common organic solvents and incomplete stereochemistry (Table S1). Since curation practices might vary between researchers, this undermines fair comparisons across machine learning methods.

While best practices for molecular data preprocessing exist,^1,3,5^ they are not consistently applied in practice. The interdisciplinary nature of the field^8^ means that not all researchers and reviewers are familiar with these conventions, and common tooling^9,10^ is flexible, rather than prescriptive. For example, it might not be obvious that molecular data often violate the assumption of independence in data splitting due to the presence of analogue series and shared scaffolds. In this context, standardizing approaches for data curation holds great potential to make studies more consistent, transparent, and reproducible. Large language models (LLMs) offer an appealing solution through conversational interfaces that could democratize access to rigorous cheminformatics workflows without requiring expertise in both programming and chemistry.^11^ However, standard LLMs are not equipped to be applied directly to molecular data and lack the domain constraints necessary to enforce the required level of rigor. Notable efforts to address this include ChemCrow^11^ and ChatInvent,^12^ which augment LLMs with chemistry tools to execute multistep workflows. However, while these systems emphasize task execution, they do not address the data-centric steps that underpin the molecular machine learning studies.

In answer to these limitations, we present ChemLint (Figure 1) as a bridge between robust cheminformatics tooling and conversational AI through the Model Context Protocol^13^ (MCP). MCP is a lightweight communication standard that provides a layer for connecting LLM clients to external tools hosted by a (local) server, without requiring tight integration or custom plugins. An MCP server exposes tool specifications (including input schemas) to an LLM client and returns machine-readable outputs when the client delegates a task to the server. This enables deterministic execution of specific operations that require computation, limiting LLM “hallucinations.” Because MCP is client-agnostic, any MCP-compatible conversational interface (*e*.*g*., Claude or ChatGPT) can be used as the front end to interact with molecular data.

**Figure 1:**
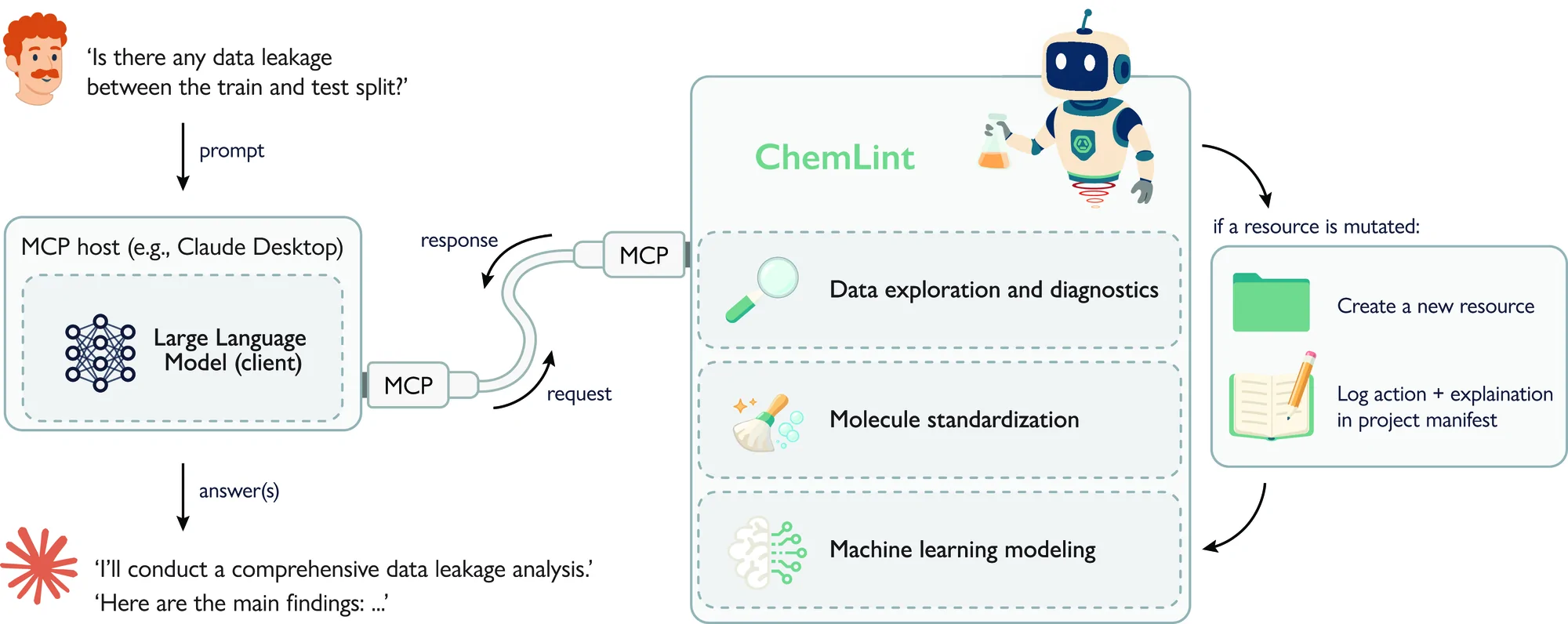
Overview of ChemLint. An MCP-enabled LLM client (e.g., Claude Desktop) sends prompts to the ChemLint server, which performs cheminformatics tasks (data exploration/diagnostics, molecule standardization, and machine learning modeling) and returns results. Any operation that mutates a resource creates a new version, which ChemLint logs in a project manifest, alongside an explanation of the action that created it.

ChemLint uses MCP to connect conversational user queries to a local suite of cheminformatics and molecular machine learning tools. The client LLM is used for interaction, planning, and interpretation, while all molecule-level operations are carried out by tooling with explicit constraints. In addition, molecular data remain local (the client exchanges only structured metadata and references to local assets), and all operations are automatically tracked in a project manifest that records key inputs and parameters, creating an audit trail for reproducibility of experiments. This design provides guardrails without sacrificing exploratory freedom; users interact through natural language, while ChemLint constrains molecular operations to deterministic tooling, reducing the risk of silent data-handling errors and improving adherence to cheminformatics best practices. Throughout, ChemLint applies sensible best-practice defaults unless the user explicitly requests an alternative.

ChemLint provides a complete workflow for rigorous molecular machine learning, emphasizing the data quality and understanding. This is achieved through three layers of functionality: (1) exploratory data analysis tools and diagnostics to characterize data sets and identify data quality issues (e.g., chemically invalid SMILES, data leakage across splits), (2) systematic tools for data handling (e.g., standardization pipelines and conflict resolution), and (3) machine learning tools for building baseline models. Unlike conventional scripting or pipeline-based environments, ChemLint formalizes molecular data operations as explicitly defined, inspectable, and LLM-accessible tools, enabling conversational workflows in which preprocessing decisions become structured, auditable, and reproducible rather than implicitly encoded in user-defined code. In what follows, we introduce the functionality of the tools and showcase how ChemLint could be used for conversational cheminformatics, through several examples.

## Features

ChemLint’s functionality is organized around three pillars that mirror a typical molecular machine learning workflow (Figure 1): (1) data exploration and diagnostics, (2) molecule standardization, and (3) machine learning modeling. A cross-cutting reproducibility system underpins all three.

### Data Exploration and Diagnostics

#### Quality Assessment and Reporting

ChemLint provides exploratory data analysis tools (clustering, visualization, and similarity calculations) and systematic diagnostic capabilities to help users understand their data. While ChemLint typically follows a modular approach, we implemented three complementary standardized diagnostic reports that expose critical data quality issues that are easily overlooked.

As a primary diagnostic tool, ChemLint can write a report based on a broad range of data checks covering basic data set statistics, molecular validity, physicochemical properties, statistical distributions, and structural features. Additionally, the report identifies violations of Lipinski’s Rule of Five,^14^ Veber rules,^15^ and Quantitative Estimate of Drug-likeness (QED)^16^ thresholds and applies outlier detection (using the IQR method). For data sets with activity labels, the system assesses label distributions, calculates property–activity correlations, and identifies potential structure–activity relationships. Structural analysis includes scaffold diversity metrics, functional group distributions, stereochemistry completeness, charge state analysis, and detection of salts, fragments, and special features (organometallics, isotopes, macrocycles). The report concludes with prioritized recommendations for data cleaning. In our experience, this analysis routinely identifies several distinct quality issues, even in established benchmark data sets (Table S1).

A second report targets data splitting, where subtle leakage is particularly easy to miss. Random splitting of molecular data sets risks data leakage when structurally similar molecules appear in both training and test sets. Although acceptable for lead optimization where interpolation is the goal, this inflates performance estimates in the more common scenario of benchmarking generalization to novel chemical space. To detect such issues, ChemLint can compile a specialized split quality report that performs eight systematic checks to detect potential issues with train/test splits. It detects exact duplicate SMILES, similarity-based “leakage” (high Tanimoto similarity pairs), scaffold overlap (shared Bemis–Murcko scaffolds^17^), stereoisomer/tautomer variants, differences in physicochemical property distributions, differences in label distributions, and differences in the functional groups present. All steps are accompanied by the appropriate statistical tests when possible (e.g., Kolmogorov–Smirnov tests for regression label bias or Chi-square for classification label bias). The report assigns a severity level (“OK”, “low”, “medium”, “high”, “critical”) to each issue and highlights specific examples.

Finally, a dedicated scaffold analysis report provides detailed chemical diversity assessment using five complementary analyses: computing overview statistics (total scaffolds, diversity ratio, coverage statistics), analyzing scaffold distributions (using Shannon entropy and Gini coefficient), highlighting the most frequent scaffolds, finding structural outliers based on substructure similarity, and an activity enrichment analysis by identifying privileged/inactive scaffolds.

#### Label Quality

Experimental bioactivity data inevitably contains measurement errors, missing values, technical replicates with outliers, and conflicting measurements for identical molecules. However, many published studies handle these issues *ad hoc* or ignore them entirely. ChemLint provides systematic tools for identifying and resolving label quality issues. Besides the removal of missing values, statistical outliers can be identified using multiple methods (Z-score, modified *Z*-score, IQR, Grubbs’ test, generalized Extreme Studentized Deviate) with client-configurable thresholds. For duplicate molecules with conflicting labels (e.g., aggregated stereoisomers after molecule standardization), ChemLint can determine whether conflicts represent genuine measurement variability or systematic disagreement through statistical testing. Users can then resolve duplicates with several merge strategies (majority vote, mean, or median) or drop conflicting entries entirely.

### Molecule Standardization

#### Structure Standardization

Problematic SMILES representations are a pervasive source of error in molecular machine learning.^1,3^ Some data sets might, for example, contain seemingly distinct SMILES strings that are identical under standardization. This prevents meaningful cross-study comparisons due to different curation strategies and injects unwanted chemical bias in holdout data sets. To ensure consistent handling of molecular structures, ChemLint contains an extensive set of standardization operations together with comprehensive guidelines on how the client should use them. Additionally, we implemented a default 11-step SMILES standardization protocol that covers most modeling needs (when necessary, clients will ask users for important standardization decisions, see Figure S1):

(1) SMILES canonicalization: Converts SMILES strings to their canonical form using RDKit^9^ and detects chemically invalid structures^9^ early on.
(2) Salt removal: Eliminates common counterions (Na^+^, Cl^–^, Mg^2+^, etc.) using configurable SMILES Arbitrary Target Specification (SMARTS) patterns.
(3) Solvent removal: Strips cocrystallized solvent molecules (water, ethanol, DMSO, etc.).
(4) Defragmentation: It keeps the parent molecule (the largest fragment) when more than one fragment is present.
(5) Functional Group Normalization: Standardization of nitro groups, N-oxides, azides, diazo compounds, sulfoxides, and phosphates.
(6) Reionization: Restores chemically appropriate charge states for zwitterions and multi-ionizable compounds.
(7) Charge neutralization: Converts ionizable groups to neutral forms (e.g., protonated amines to amines) with SMARTS patterns.
(8) Isotope removal (optional): It removes isotopic labels when not scientifically relevant.
(9) Metal disconnection (optional): Disconnect metal–ligand coordinate bonds in organometallic complexes.
(10) Tautomer canonicalization (optional): Standardizes keto–enol and other tautomeric forms to canonical representations.
(11) Stereochemistry handling: Keeps, assigns, or flattens stereochemical information.

#### Molecular Featurization

After standardization, molecules can be converted to numerical representations for similarity calculations, clustering, and modeling. ChemLint supports 210+ RDKit 2D descriptors (MW, LogP, TPSA, etc.) and six fingerprint types covering circular neighborhoods (extended connectivity fingerprints [ECFPs][Bibr ref18]), topological paths (atom pairs,^19^ topological torsions^20^), substructure patterns (molecular access system [MACCS],^21^ RDKit^9^), and pharmacophore features (chemically advanced template search [CATS][Bibr ref22]). Moreover, the platform supports typical representations for sequence-based deep-learning approaches, as it provides SMILES tokenization tools that can discover and count all tokens present in a data set and flag molecules that contain out-of-vocabulary tokens.

### Machine Learning

#### Data Splitting and Leakage Prevention

ChemLint supports four splitting strategies: (1) random splitting, (2) stratified splitting (preserves label distribution), (3) scaffold-based splitting (assigns all molecules sharing the same scaffold to the same split), and (4) cluster-based splitting (assigns entire clusters to splits). For cluster-based splitting, users can choose from five clustering algorithms (DBSCAN, hierarchical, spectral, *k*-means, and Butina^23^) and all available molecular representations. In our experience, the stricter splitting strategies (scaffold- and cluster-based) often reduce accuracy by 10–30% compared to random splits (Table 3), revealing a more realistic estimation of predictive performance on structurally novel molecules.

#### Model Training and Evaluation

ChemLint provides 33 classical machine learning algorithms spanning classification and regression (Table 1). To ensure robust performance estimation, multiple cross-validation strategies are supported (*k*-fold, stratified, scaffold-based, cluster-based, Monte Carlo, and leave-p-out), with entire cross-validation ensembles available for predictions and evaluation. These ensembles provide standard deviations across folds that quantify model stability, while select algorithms offer additional uncertainty quantification through Bayesian ensemble variants that compute the prediction standard deviation or ensemble entropy. Models can be evaluated using diverse metrics appropriate to the task (e.g., accuracy, ROC AUC, *R* ^2^, RMSE) and optimized through grid search over hyperparameter spaces. This combination of algorithms, robust validation, uncertainty quantification, and flexible evaluation enables users to quickly establish baseline, compare molecular representations, and perform predictions on new molecules. We did, however, not intend ChemLint to be a tool to develop new state-of-the-art models or deal with long and/or complex training procedures and thus do not support deep-learning approaches as of now.

**Table 1:** Machine Learning Methods Supported by ChemLint^a^

algorithm	supported tasks	uncertainty support	key characteristics
Random Forest	both	yes	ensemble of decision trees, handles nonlinear relationships, robust to overfitting
Gradient Boosting	both	yes	sequential ensemble learning, often achieves highest accuracy
Logistic Regression	classification	yes	linear, probabilistic, highly interpretable
Ridge Regression	regression	no	linear, L2 regularization, stable and fast
Lasso	regression	no	L1 regularization, automatic feature selection
Elastic Net	regression	no	combined L1 + L2 regularization
Support Vector Machine	both	no	kernel methods, margin optimization for classification/regression
K-Nearest Neighbors	both	no	instance-based learning, distance-weighted predictions
Decision Tree	both	yes	single interpretable tree structure
AdaBoost	both	yes	adaptive boosting, focuses on hard examples
Extra Trees	both	yes	extremely randomized trees, faster than Random Forest
Naive Bayes	classification	no	probabilistic classifier based on Bayes’ theorem
Linear Discriminant Analysis	classification	no	linear classifier with dimensionality reduction
Stochastic Gradient Descent	both	no	online learning, scalable to large data sets

a Supported tasks are either regression or binary classification. For some methods, uncertainty estimation is enabled by ensembling models with different random seeds. Several methods are provided as separate regression and classification variants, which are counted individually (33 in total).

### Reproducibility and Data Provenance

An LLM that can perform many mutating operations on a molecular data set on its own account (e.g., removing outliers) poses clear challenges for reproducibility. For that reason, all resources are traced with a unique identifier in a project-based manifest that logs all files and their history. Every operation performed by the server that mutates a resource creates a new resource (keeping the old one) and is automatically logged in a project manifest (i.e., an LLM lab journal). Traceability is achieved by forcing the client to provide a name for the new resource and a short explanation for the performed operation. Since this manifest (alongside all data files) is locally stored in a working directory and can be accessed by the client, both the user and the client can back-track every individual resource. While the recorded operations and their results are fully reproducible, the host model’s natural-language explanations may vary between runs.

## Examples

### Example 1: Quality Audit of Popular Benchmark Data Sets

As a first demonstration, we used ChemLint to evaluate the quality of seven popular single-task benchmark data sets from MoleculeNet^7^ using a single conversational prompt: “Check the data quality of dataset.csv.” (Figure S2).

The results (Table S1) reveal several concerns. Some data sets contain chemically invalid SMILES strings outright (BBBP: 11, HIV: 7, ClinTox: 4). Many entries include salt counterions or solvent fragments (up to 7.5% in HIV). Since these benchmark tasks aim to model structure–activity relationships of parent compounds, not formulation metadata, such fragments introduce inconsistent representations and noise. Charged species are similarly prevalent: over 55% of BACE and 60% of ClinTox molecules carry formal charges. While charges can reflect genuine assay conditions or permanent ions, in data sets compiled from heterogeneous sources, they may reflect assay conditions or specific encoding conventions that complicate QSAR modeling.

Perhaps most concerning is the incomplete specification of the stereochemistry. In the BBBP, for example, only 66% of chiral centers and 21.5% of stereogenic double bonds are fully specified. Modeling data sets with partially defined stereochemistry risks introducing systematic noise, as structurally distinct stereoisomers may be treated as identical or vice versa. In such cases, removing all stereochemical assumptions is often the most-defensible choice. Notably, while none of these data sets contain duplicate SMILES in their original form, flattening stereochemistry and canonicalizing tautomers reveals substantial hidden redundancy: groups of structural isomers that collapse to identical canonical representations, sometimes with conflicting labels (Table S1, rightmost column).

We then asked ChemLint to clean each data set: “Clean this data set so it is ready for machine learning (do not split the data yet). After cleaning, run another data quality analysis.” (Figure S3). Since all data sets except HIV are compiled from multiple original sources, we let the client neutralize charges, remove fragments, and flatten stereochemistry in all data sets, as it is unlikely that these molecular details reflect consistent experimental conditions across the original sources.

The effects were substantial (Table 2). Neutralization alone reduced charged species in BACE from ∼56% to ∼2% and in ClinTox from ∼60% to ∼8%. Across all seven data sets, between 3 and 238 molecules were discarded due to chemical invalidity, conflicting duplicate labels, or other irrecoverable issues. After standardization, all data sets were free of invalid molecules, salts, and fragments, though residual charges remain where they reflect permanent ionic species.

**Table 2:** Data Quality after Molecule Standardization by ChemLint^a^

data set	*N*	discarded	invalid molecules	charged	fragments or salts
BACE	1447	66	0	1.9%	0.00%
BBBP	1922	128	0	3.2%	0.00%
ClinTox	1340	144	0	8.1%	0.00%
Delaney	1114	14	0	5.6%	0.00%
FreeSolv	639	3	0	5.9%	0.00%
HIV	40,889	238	0	13.1%	0.00%
lipophilicity	4092	108	0	2.4%	0.00%

a SMILES strings were canonicalized, de-salted, de-fragmented, ionizable groups were neutralized, and stereochemistry was flattened. Molecules that were chemically invalid were discarded. For groups of structural isomers with identical classification labels, one instance was kept. For groups of structural isomers with statistically indistinguishable regression labels, the mean label of the group was used. Structural isomers with conflicting labels were discarded altogether.

### Example 2: Data Split Quality Analysis of Popular Benchmark Data Sets

As a second demonstration, we used ChemLint to analyze the quality of predefined data splits provided by MoleculeNet.^7^ As a follow-up prompt in the conversations from Example 1, we asked: “Check for data leakage between the following three predefined data splits: ...”, supplying the file paths for each split.

ChemLint computed scaffold overlap between train and test sets, checked for the presence of stereoisomers and tautomers across splits, flagged highly similar molecules (Tanimoto similarity ≥0.9 on 2048-bit ECFPs), and performed statistical testing on label and physicochemical property distributions. It returned a report listing all major issues with explanations and recommendations for model development. For example, for the Lipophilicity data set, it concluded: “The scaffold splitting approach provides the most reliable evaluation framework with complete structural separation and well-matched distributions. Fingerprint splitting offers interesting insights into model extrapolation but suffers from significant domain shift. Random splitting should be avoided for model evaluation due to severe structural leakage.” In all cases, ChemLint recommended against using the random splits, for example, warning for ClinTox that random splitting would “give misleadingly optimistic results” due to severe structural leakage.

We compiled the key leakage metrics reported by ChemLint in Table 3. For random splits, ChemLint identified scaffold overlap between train and test sets ranging from 42.5% to 63.6%, along with stereoisomer, tautomer, and near-duplicate leakage across several data sets. For scaffold-based splits, ChemLint confirmed that most leakage was resolved but noted that highly similar molecules can still end up in both splits and that tautomerization can occasionally alter the Bemis–Murcko scaffold, allowing tautomer pairs to leak across sets. For fingerprint-based splits, ChemLint reported no near-duplicate leakage but flagged residual scaffold overlap, reflecting the fact that these two similarity measures capture different structural aspects. It also caught a subtle artifact in the FreeSolv data set, where fingerprint-based splitting placed most aliphatic molecules in the test set, creating a strong out-of-distribution scenario. The client warned: “Fingerprint splitting creates an unusual distribution where test molecules are acyclic/aliphatic with high flexibility (3.72 rotatable bonds vs 1.38 in train).”

**Table 3:** Test Set Characteristics for Different Splitting Approaches of Seven Popular Benchmark Data Sets as Analyzed by ChemLint^a^

splitting method	data set	*N* train (test)	scaffold overlap	stereoisomer overlap	tautomer overlap	highly similar molecules	ROC AUC	RMSE
random	BACE	1210 (152)	47.1%	1	0	13	0.88 ± 0.01	
	BBBP	1631 (204)	42.5%	13	11	16	0.91 ± 0.02	
	ClinTox	1184 (148)	46.5%	14	10	16	0.66 ± 0.03	
	Delaney	902 (113)	58.1%	2	1	10		0.64 ± 0.00
	FreeSolv	513 (65)	63.6%	1	0	6		0.46 ± 0.02
	HIV	32,896 (4112)	48.0%	0	4	173	0.77 ± 0.01	
	lipophilicity	3360 (420)	46.5%	18	3	31		0.70 ± 0.01
scaffold	BACE	1210 (152)	0.0%	0	0	2	0.73 ± 0.01	
	BBBP	1631 (204)	0.0%	0	1	0	0.67 ± 0.01	
	ClinTox	1184 (148)	0.0%	0	0	0	0.66 ± 0.08	
	Delaney	902 (113)	0.0%	0	0	2		0.82 ± 0.01
	FreeSolv	513 (65)	0.0%	0	0	1		0.86 ± 0.01
	HIV	32,896 (4112)	0.0%	0	8	29	0.77 ± 0.01	
	lipophilicity	3360 (420)	0.0%	0	0	21		0.77 ± 0.01
fingerprint	BACE	1210 (152)	3.2%	0	0	1	0.73 ± 0.06	
	BBBP	1631 (205)	4.6%	0	0	0	0.37 ± 0.06	
	ClinTox	1184 (148)	5.8%	0	0	0	0.56 ± 0.10	
	Delaney	902 (114)	28.1%	0	0	0		1.23 ± 0.04
	FreeSolv	513 (65)	100.0%	0	0	0		1.36 ± 0.02
	HIV	32,896 (4112)	10.9%	0	0	0	0.56 ± 0.03	
	lipophilicity	3360 (420)	4.4%	0	0	0		0.84 ± 0.01

a Data splits are provided by MoleculeNet.^7^ Scaffold overlap refers to the percentage of scaffold from the test set that occur in the training set. Stereoisomer overlap measures the presence of structural isomers after flattening stereochemistry. Tautomer overlap measures the number of structural isomers after canonicalizing tautomers. Test molecules are considered highly similar to training molecules (Tanimoto coefficient ≥0.9 on 2048-bit ECFPs, radius 2). Reported ROC AUC and RMSE values are means ± standard deviations across cross-validation folds.

Finally, we compared modeling performance across splitting methods by prompting: “train a random forest model with standard hyperparameters and 5-fold Monte Carlo cross-validation.” Results (Table 3) emphasized that “Random splitting consistently inflates performance.” Given that numerous studies have evaluated model performance on these exact splits without discussing their limitations, these issues are evidently easy to miss.^7,24,25^ Our results illustrate how conversational cheminformatics can help researchers identify and understand such splitting issues with minimal effort.

### Example 3: From Data to Report

As a third demonstration, we used ChemLint to execute a complete modeling pipeline from raw data to an evaluation report. Claude Desktop with Claude Sonnet 4.5 was used as the client, and we provided the following prompt:

“For a drug discovery project, I want to know if the molecules I’m working with can pass the blood brain barrier. Train a robust predictive model based on this raw data set ’/moleculenet_data/bbbp.csv’ and evaluate it critically.”

The client proceeded to use ChemLint to import the data, perform exploratory data analysis, standardize the SMILES strings, and remove duplicates. It then split the data by scaffolds (80/20) and trained a Random Forest classifier with 5-fold cross-validation, achieving an ROC AUC of 0.93 ± 0.01 on the held-out test set.

To test the reproducibility of the manifest system, we created a new client instance and asked it to reproduce the experiment without access to the original prompt: “Check this old manifest ‘···/manifest.json’ and redo all the experimental steps exactly. You should make a new project and manifest to track everything.” The client executed all steps in the same order with identical parameters and achieved the same test set performance.

Finally, we asked the client to generate a methods section: “Write a Materials and Methods section suitable for publishing in a scientific journal. I am tight on words so it should be to the point.” The client automatically used ChemLint to consult the project manifest, after which it produced the text shown in Box [Boxed-text box1]. Manual verification of every recorded step confirmed the accuracy of the generated method section.

1. Material and Methods section written by Claude Sonnet 4.6 and ChemLint. Model output is shown without editing.

**Materials and Methods**

***Data set and data curation***. The BBBP data set (2050 compounds, binary BBB-permeability labels) was obtained from MoleculeNet [REF]. SMILES were standardized using an 11-step pipeline implemented in ChemLint [REF]: canonical SMILES generation, salt and solvent removal, defragmentation (largest fragment retained), functional group normalization, deionization, charge neutralization, isotope removal, tautomer canonicalization, and stereochemistry flattening. Structures failing final validation were discarded (2039 retained; 99.5%). Duplicate structures were identified on canonical SMILES; exact-label duplicates were collapsed to a single entry, and conflicts unresolvable by binomial test (α = 0.05) were removed entirely, yielding a final curated set of 1922 compounds.

***Data set splitting***. Compounds were split into training (80%, *n* = 1557) and test (20%, *n* = 365) sets using Bemis–Murcko scaffold-based partitioning [REF], with no scaffold overlap between sets. Acyclic compounds (*n* = 96) were assigned to training.

*Modeling*. Molecules were featurized as ECFP4 fingerprints (radius = 2, 2048 bits), and a Random Forest classifier (scikit-learn [REF], default hyperparameters) was trained using 5-fold scaffold cross-validation, all via ChemLint [REF]. All experiments used a fixed random seed (42).

While LLM clients may behave differently across prompts (e.g., Figures S2 and S3, the client changed its mind on zwitterion handling), this example illustrates that establishing a clear audit trail through the manifest system enables reproducibility even in a conversational setting.

## Implementation

ChemLint exposes approximately 150 tools to MCP-compatible LLM clients using the mcp-cli package (https://github.com/philschmid/mcp-cli). Each tool corresponds to a single well-scoped molecular data operation. Computational work is delegated to established libraries, primarily RDKit^9^ for molecular processing, pandas for data set handling, scikit-learn^26^ for modeling and statistical routines, and SciPy for hypothesis testing. The main contribution is therefore not algorithmic but consists of a constrained and auditable workflow layer that makes common cheminformatics best practices easy to apply from a conversational interface. Because all tool schemas and their documentation are exposed to the host model, they occupy space in its context window: models with large context windows handle the full tool set comfortably, whereas smaller models may require more specific prompting as a conversation grows. We minimized the documentation redundancy to limit this overhead.

The tool interface is intentionally granular. ChemLint provides composable operations (e.g., SMILES canonicalization, salt removal, filtering, splitting, diagnostics) that can be combined into workflows appropriate for a given data set and modeling objective. This reduces the risk of hidden preprocessing choices and makes each step explicit and inspectable. Even higher level functions, such as the standardization pipeline, are treated as a chain of separate functions and produce intermediate SMILES and per-molecule status annotations at each stage. These are stored as data set columns so that failures are transparent, including which molecules were rejected at which step and why. This is intentionally verbose, since diagnosing rejection causes often matters more than obtaining a single final “cleaned” data set.

Resource management is handled by assigning a unique identifier that is appended to a client-provided filename (e.g., cleaned_data_A3F2B1D4.csv), allowing intermediate results to be referenced unambiguously. For each artifact, the manifest records the resource type, timestamp, which tool created it plus its input parameters, and a short client-provided explanation. Any tools added to ChemLint in the future will be logged in this same manner.

The current scope focuses on 2D molecular representations and workflows that are typical of QSAR modeling. Functionality such as 3D conformer generation, quantum chemistry, and deep-learning model training is deliberately out of scope in the present version to keep ChemLint focused on data quality, diagnostics, and reproducible evaluation rather than acting as a general-purpose modeling environment. An important direction for future work is aligning validation strategies with how the model will ultimately be used, which the current version does not yet automate.

## Conclusion

Agentic systems such as ChemCrow^11^ and ChatInvent^12^ have shown that LLMs can coordinate end-to-end molecular design and synthesis workflows. In parallel, MCP-based tools have emerged as a flexible way to connect LLMs to scientific software, with early applications in polymer design,^27^ protein modeling,^28^ and bioinformatics.^29^ ChemLint fits naturally into these ecosystems by focusing on a step that is often overlooked: enabling researchers to interrogate existing data sets and make defensible choices about standardization, data splitting, and evaluation before any model training. While community resources such as MoleculeNet,^7^ Therapeutics Data Commons (TDC),^30^ and DeepChem^31^ provide data sets and modeling infrastructure, they often leave these preprocessing decisions implicit and user-dependent. ChemLint adds a conversational interface that surfaces these decisions explicitly and complements the existing benchmarks and toolkits.

ChemLint was developed to make rigorous cheminformatics workflows easier to apply, reproduce, and interpret. By pairing a conversational interface with a constrained, tool-based Application Programming Interface (API), the system supports data set exploration, systematic diagnostics of common data quality issues, and the application of best-practice strategies without relying on ad hoc scripts or undocumented manual steps. All operations are executed through local tooling and recorded in a project manifest, producing a clear audit trail that documents how each data set artifact, split, and model was created. ChemLint currently addresses reproducibility and mitigates hallucination by grounding LLM actions in deterministic tools, while implementing the standard evaluation practices expected of a machine learning workflow. Evaluation tailored to the user’s specific task remains a key open challenge and a natural direction for future work.

Beyond automation, ChemLint is designed to make the assumptions and trade-offs in molecular data handling visible. Diagnostic reports, standardization annotations, and split quality checks aim to highlight issues that are easy to overlook but can change downstream modeling results. Together, these features position ChemLint as a practical workbench for conversational cheminformatics. While ChemLint does not replace conventional modeling environments, nor eliminate the need for expert judgment, it lowers the entrance barriers into the field and provides a structured framework that reduces ambiguity in data handling decisions—ultimately improving the transparency and reproducibility of molecular machine learning workflows.

## Supplementary Material



## Data Availability

ChemLint is available at https://github.com/molML/ChemLint as an open-source package. Proprietary MCP-enabled clients, such as Anthropic’s Claude, OpenAI’s ChatGPT, and Google’s Gemini are available through their respective subscription plans. Open-source clients such as 5ire (https://5ire.app) provide free alternatives and can be paired with locally hosted models such as Ollama (https://ollama.com), though setup requires additional configuration compared to integrated platforms.

## Notes

Data quality analysis of the seven benchmark data sets in their original form (Table S1); screenshots of an MCP client interacting with the user (Figure S1); screenshot of performing data quality analysis on a benchmark data set (Figure S2); and screenshot of cleaning a benchmark data set (Figure S3) (PDF)
