# Supplementary material for: ChemLint: Conversational Cheminformatics with Large Language Models
Source: J Chem Inf Model. 2026 Aug 28;66(19):12568–75. doi: 10.1021/acs.jcim.6c00638 (PMC13663861; doi:10.1021/acs.jcim.6c00638)
Supplement: Supplementary file 1 [file ci6c00638_si_001.pdf]

Supporting Information

ChemLint: Conversational Cheminformatics with Large Language Models

Derek van Tilborg[1*] and Francesca Grisoni[1*]

[1]Institute for Complex Molecular Systems (ICMS), Department of Biomedical Engineering, Eindhoven University of Technology, PO Box 513, 5600 MB Eindhoven, The Netherlands.

*Corresponding authors: d.w.v.tilborg@tue.nl, f.grisoni@tue.nl

Supplementary Table S1: Data quality as analyzed by ChemLint. Datasets are analyzed in their original form (downloaded from MoleculeNet[7]) without additional curation. Molecules are considered structural isomers if they have identical canonical SMILES strings after flattening stereochemistry and standardizing tautomers.

Dataset	N	Invalid molecules	Charged	Fragments or salts	Chiral centers (specified)	Stereogenic double bonds (E/Z specified)	Structural isomer groups
BACE	1,513	0	55.92%	0.00%	3150 (25.5%)	97 (29.9%)	45
BBBP	2,050	11	5.74%	5.12%	4425 (66.0%)	726 (21.5%)	92
ClinTox	1,484	4	60.20%	0.94%	3731 (82.1%)	537 (37.2%)	80
Delaney	1,128	0	5.23%	0.00%	701 (0.0%)	154 (3.9%)	13
FreeSolv	642	0	5.92%	0.00%	87 (98.9%)	36 (27.8%)	3
HIV	41,127	7	12.78%	7.51%	49613 (0.0%)	13481 (0.0%)	181
Lipophilicity	4,200	0	2.36%	0.02%	2530 (72.9%)	192 (39.1%)	82

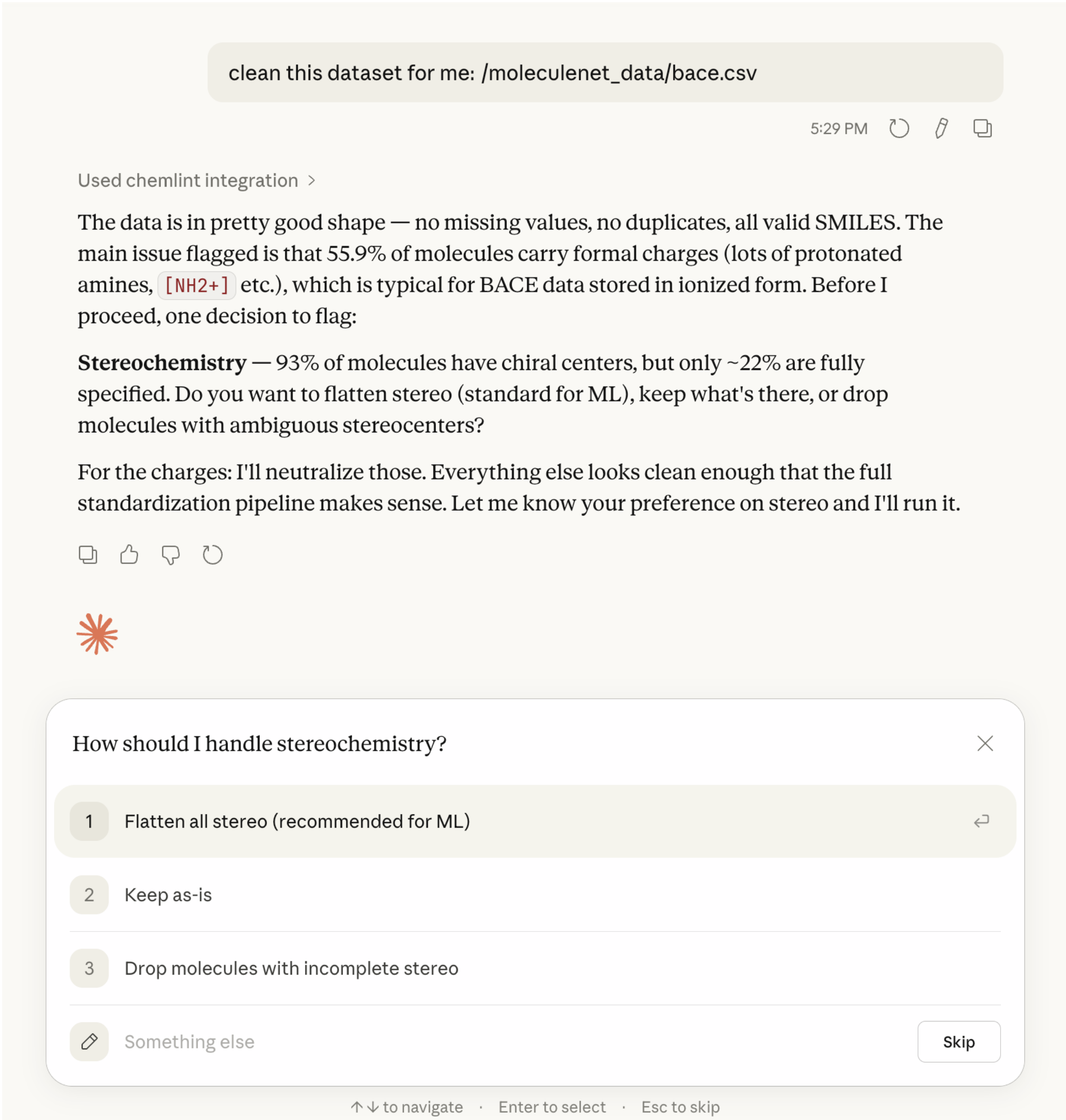

Supplementary Figure 1: Screenshot of a client (Claude Sonnet 4.6) interacting with the user in Claude Desktop (Anthropic, claude.ai). Use of Claude Desktop is for academic illustration only and does not imply affiliation with or endorsement by Anthropic. Other clients with MCP-support can be used with ChemLint but might behave differently.

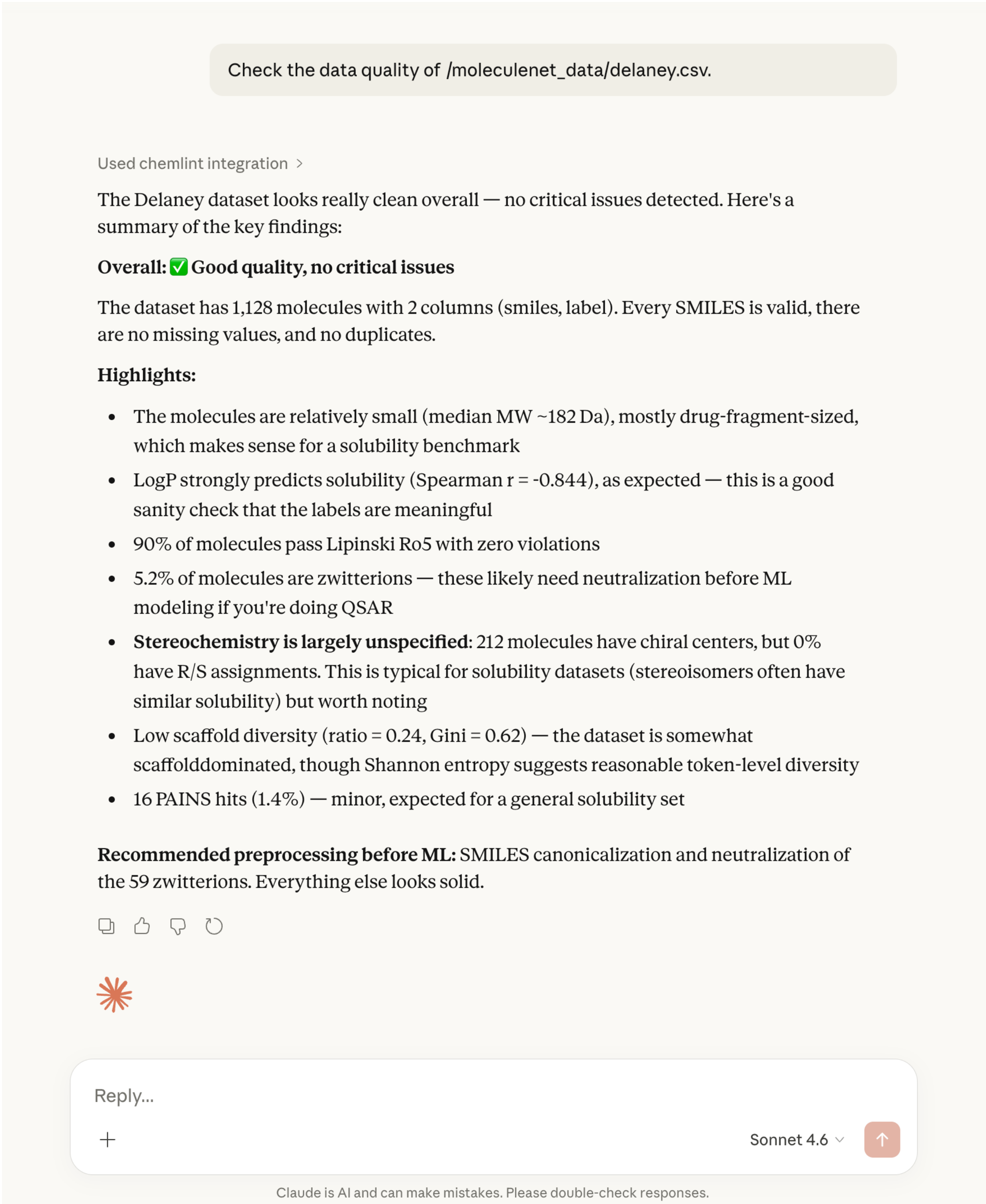

Supplementary Figure 2: Screenshot of a client (Claude Sonnet 4.6) using ChemLint to perform data quality analysis on the Delaney benchmark dataset. Use of Claude Desktop is for academic illustration only and does not imply affiliation with or endorsement by Anthropic.

Clean this dataset so it's ready for machine learning (don't split the data yet). After cleaning, run another data quality analysis.

I'll run a full cleaning pipeline on the Delaney dataset. Based on the quality report, the steps are: canonicalize SMILES, standardize (salt removal, normalization, reionization, neutralization), deduplicate, and validate. Let's go.

Default SMILES standardization pipeline dataset >

All 1128 molecules passed standardization. Now let's keep only the clean columns, then check for duplicates that may have emerged post-standardization.

Used chemlint integration >

Good — 13 duplicate groups emerged after standardization (stereoisomers that collapsed to the same structure). Let's deduplicate them.

Deduplicate dataset >

1128 → 1114 molecules after deduplication. Now let's rename the column to `smiles` for cleanliness and run the final quality report.

Used chemlint integration >

Now let's run the full quality analysis on the cleaned dataset.

Data quality analysis >

All clean. Here's a summary of what was done and what the post-cleaning report shows:

Cleaning pipeline summary

The dataset went from **1,128 → 1,114 molecules** through four steps: full SMILES standardization (canonicalization, salt/solvent removal, defragmentation, functional group normalization, reionization, neutralization, isotope removal, tautomer canonicalization, and stereo flattening), followed by deduplication. The 14 removed entries were stereoisomers that collapsed to the same flat structure after standardization — 7 had nearly identical labels (kept first), 5 had consistent labels that were averaged, 1 was highly conflicting (dropped), and 1 was a true exact duplicate.

Before vs. after comparison

	Before	After
Molecules	1,128	1,114
Valid SMILES	100%	100%
Duplicates	0	0
Zwitterions/charged	59 (5.2%)	62 (5.6%)
PAINS	16 (1.4%)	9 (0.8%)
Unique scaffolds	268	279
Scaffold diversity ratio	0.238	0.250

Remaining considerations

The 62 charged/zwitterionic molecules (5.6%) are still present — these were kept intentionally since solubility is inherently tied to ionization state and neutralizing them would alter the physical chemistry the labels reflect. The 9 remaining PAINS hits are low enough to leave in for a general solubility benchmark. Stereochemistry is fully flattened and consistent. The dataset is ready for featurization and splitting.

The cleaned file is tracked as `delaney_clean_final_A0626BDA.csv` in the manifest.

Reply...

Sonnet 4.6

Claude is AI and can make mistakes. Please double-check responses.

Supplementary Figure 3: Screenshot of a client (Claude Sonnet 4.6) using ChemLint to clean the Delaney benchmark dataset. Use of Claude Desktop is for academic illustration only and does not imply affiliation with or endorsement by Anthropic.
